# High-throughput screening of laser additive manufactured metallic glass via ultrasonic wave

**DOI:** 10.1038/s41598-019-54293-w

**Published:** 2019-11-27

**Authors:** Linlin Zhai, Yunzhuo Lu, Xinyu Zhao, Lu Wang, Xing Lu

**Affiliations:** 1 School of Materials Science and Engineering, Dalian Jiaotong University, Dalian, 116028 People’s Republic of China; 2 Dalian Product Quality Inspection and Testing Institute Co., Ltd., Dalian, 116630 People’s Republic of China

**Keywords:** Glasses, Metals and alloys

## Abstract

Laser additive manufacturing (LAM) technology provides an opportunity to fabricate bulk metallic glasses (BMGs) without any dimensional constraint and achieve the large-scale applications of BMGs. However, flaws, such as cracks, gas porosity, and crystalline phases, are always formed accompanied by the process of LAM, which seriously worsens the mechanical and physical properties of the resulting BMGs. Here, we present a novel method that involves ultrasonic wave technique to high-throughput screen the optimum process parameters for the LAM of BMG. A parameter library, constituted by a series of rectangular BMG samples, is rapidly fabricated by the LAM method under continuously changed combinations of laser power and travel speed. The ultrasonic attenuation factor, which is sensitive to the flaws, is used as the monitor to screen the parameters of the BMGs fabricated by the LAM. Using this approach, the laser power of 1300 W and travel speed of 600 mm/min are estimated as the optimum parameter combination for the LAM of a Zr_51_Ti_5_Ni_10_Cu_25_Al_9_ (Zr51) BMG with the slightest flaws. The amorphous-phase dominated microstructure and the sufficiently high tensile strength of the subsequent fabricated large-sized Zr51 BMG sample verify this optimum parameter combination.

## Introduction

Bulk metallic glasses (BMGs), a novel class of metallic materials, have attracted great attention due to their outstanding properties such as high strength, elasticity, corrosion resistance, and unique processing capabilities^1–3^. These remarkable properties are originated from their liquid-like atomic structure, the absence of grain boundaries and dislocation, which inherits from molten liquid and is usually obtained by rapid quenching techniques^4,5^. The most common way of fabricating BMGs is the copper mould casting. However, this method can just obtain a low cooling rate on the order of 10^2^ K/s, which restricts the BMGs to a small geometrical size in order to realize fast heat dissipation and suppress the crystallization. For example, even for the world’s largest metallic glass Pd_40_Cu_30_P_20_Ni_10_, its critical diameter fabricated by this method is only 72 mm^6^. The application scope of BMGs is severely limited due to their small dimensions in the region of only tens of millimeters. As for the future, or in the near term, it is very difficult to produce large enough metallic glasses by employing rapid casting techniques. Therefore, how to break the size restriction is a key to achieve the wide applications of BMGs.

Laser additive manufacturing (LAM) technology, which builds three dimensional components by using a laser to fuse powdered materials layer by layer, offers an excellent opportunity to manufacture BMGs without dimensional constraint^7–16^. In the LAM process, metallic powders are heated and melted rapidly and periodically by a fast-moving laser beam^17^. The cooling rates of the molten metal solution can reach the values on the order of 10^3^–10^4^ K/s, which is significantly higher than the critical cooling rates required to produce amorphous structures of most BMGs^18^. However, flaws, such as cracks, gas porosity, and crystalline phases, are always formed accompanied by the process of layer-by-layer deposition, which seriously worsens the mechanical and physical properties of the resulting BMGs^9,19,20^. Thus, it is extremely necessary to inspect the flaws in the BMGs fabricated by the LAM, especially the internal defects that are difficult to detect visually. This motivates us toward a simple and direct assay for evaluating of the hidden flaws in the BMGs fabricated by the LAM.

Ultrasonic wave testing, which is a non-destructive measurement technique, provides a unique opportunity for high-throughout identifying all the internal flaws in the components^21^. During ultrasonic wave testing, the sound energy propagates through the tested components in the form of waves^22^. The flaws can be detected from the discontinuity in the wave path, since part of the energy will be reflected back by the surfaces of cracks, cavitations, and the grain boundaries of crystalline phases^23^. The ultrasonic wave can propagate a long distance along the tested structure and consequently, ultrasonic wave testing can fast and efficiently probe the flaws for the entire sample volume rather than specific to a selected local region. Then compared to the conventional flaw-detection methods that based on the metallographic examination or the scanning electron microscope (SEM) analysis, the ultrasonic wave testing is more suitable for high-throughput screening the optimum process parameters for the LAM of metallic materials.

In the present work, a novel method that involves ultrasonic wave technique is applied to high-throughput screen the optimum process parameters for the LAM of Zr_51_Ti_5_Ni_10_Cu_25_Al_9_ (Zr51) BMG, which is chosen as the model BMG material. A parameter library, constituted by a series of rectangular Zr51 BMG samples, is rapidly fabricated by the LAM method under continuously changed combinations of laser power and travel speed. The ultrasonic attenuation factor is used as the monitor to screen the parameters of the Zr51 BMGs fabricated by the LAM. Using this approach, the laser power of 1300 W and travel speed of 600 mm/min are estimated as the optimum parameter combination for the LAM of Zr51 BMG with the slightest flaws. The amorphous-phase dominated microstructure and the sufficiently high tensile strength of the subsequent manufactured large-sized Zr51 BMG sample verify this optimum parameter combination.

## Results and Discussion

### Validating the accuracy of the ultrasonic wave testing

**Figure 1:**
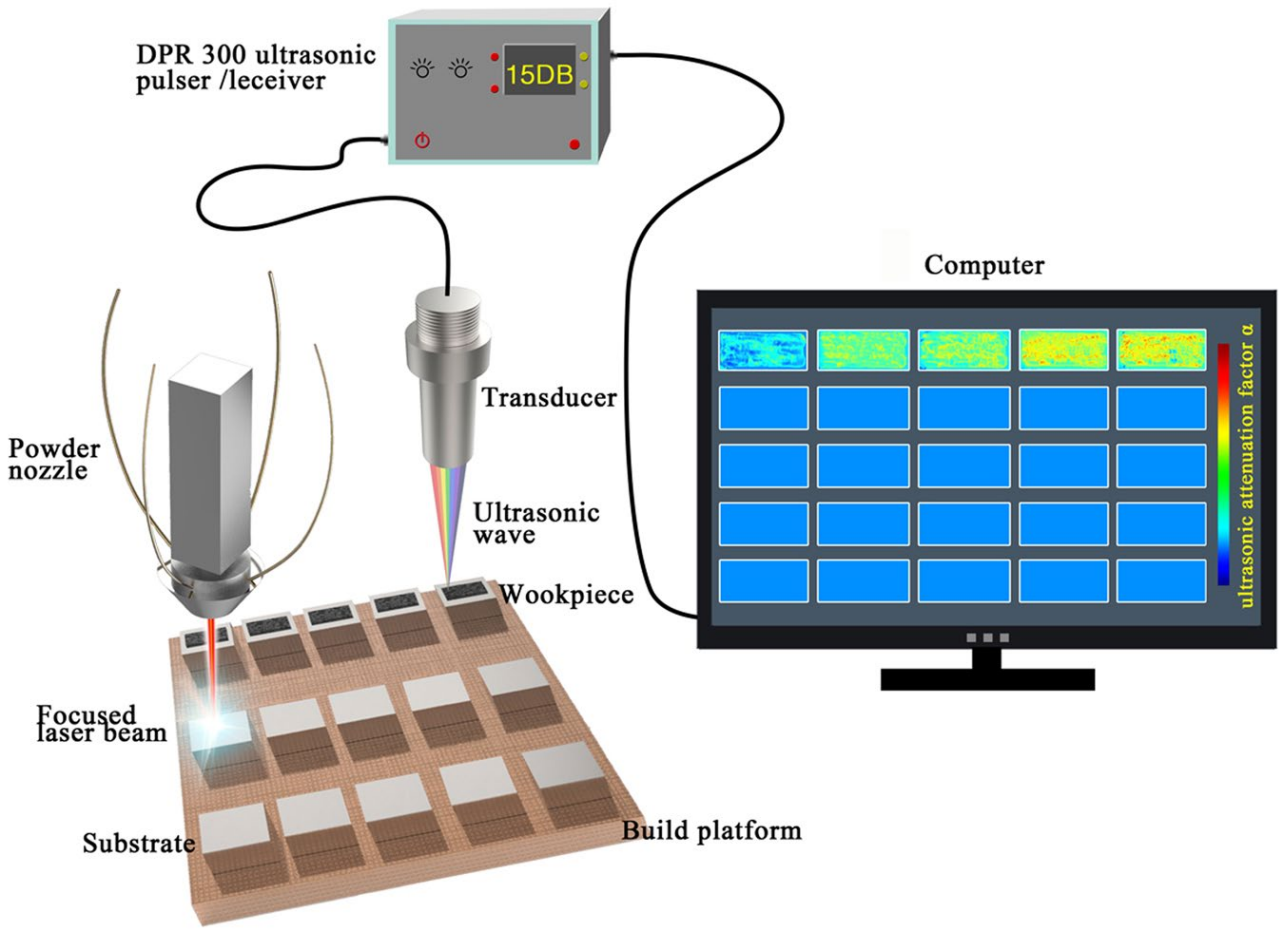
A schematic illustration of the overall experimental concept.

**Figure 2:**
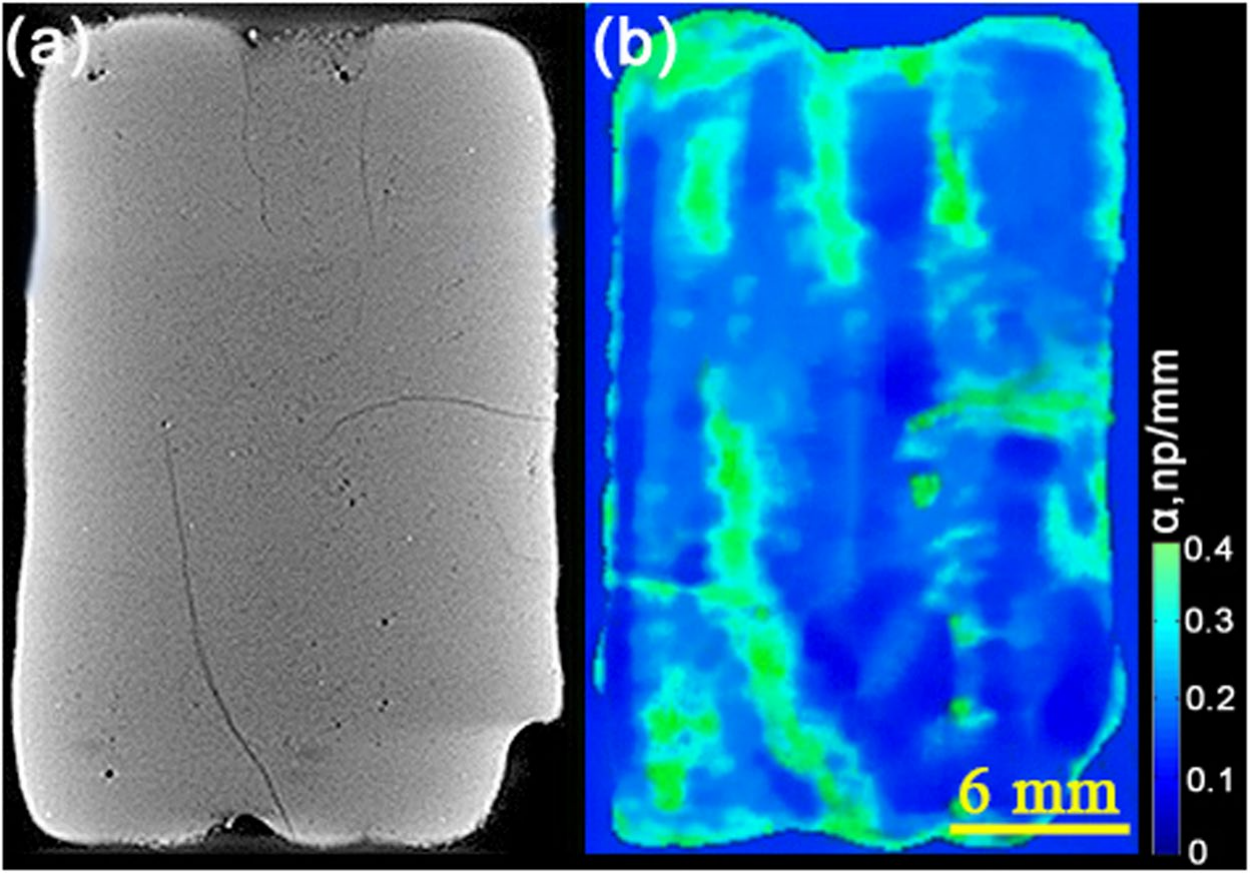
Validating the accuracy of the ultrasonic wave testing. (**a**) and (**b**) are the comparative analysis of X-ray computed tomography graphic and ultrasonic C-scan image for the same specimen. The laser power of 1200 W and travel speed of 750 mm/min are used to fabricate the specimen. The solid material and defects are respectively represented as light and dark in (**a**). The colorbar shown in (**b**) indicates the α values for the ultrasonic attenuation.

Figure 1 provides a schematic illustration of the experimental setup. To validate the accuracy of the flaw information detected from ultrasonic wave testing, a typical BMG specimen fabricated by the LAM tested by ultrasonic wave testing is also examined by X-ray Computed Tomography. The laser power of 1200 W and travel speed of 750 mm/min are used to fabricate the typical specimen. The comparative analysis of X-ray computed tomography graphic and ultrasonic C-scan image for the same specimen is shown in Fig. 2a,b, respectively. The solid material and defects are represented as light and dark in Fig. 2(a). Some internal cracks obviously are observed, furthermore, several pore can also be detected in the X-ray micro computed tomography graphic. Since part of the ultrasonic energy will be reflected back to receiver when there are pores, cracks or crystalline phases, the ultrasonic attenuation factor α, which represents the intensity of energy reflection, can be used to give a good measure of the flaw severity^24^. The colorbar shown in Fig. 2(b) indicates the α-values for the ultrasonic attenuation. Through comparing Fig. 2(a,b), it is obviously found that the dark local regions in Fig. 2(a) originated from defects exhibit relatively high α-values presented in Fig. 2(b). This evident correlation between the X-ray micro computed tomography graphic and the ultrasonic C-scan image proves that the ultrasonic is an accurate tool for the flaw evaluation in the BMG fabricated by the LAM.

### High-throughput screening of BMG fabricated by the LAM via ultrasonic wave

**Figure 3:**
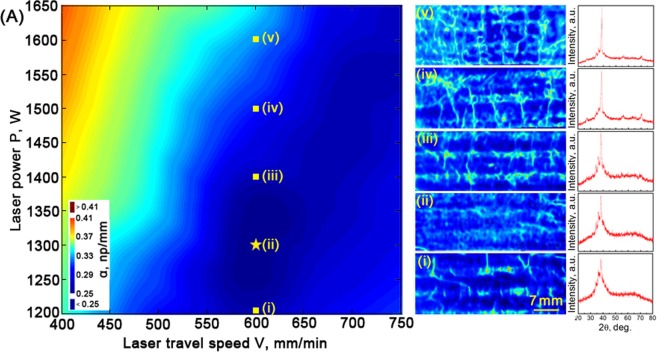
High-throughput screening of BMGs fabricated by the LAM via ultrasonic wave. (*A*) The mean α value mapping for the BMGs fabricated by the LAM from various combinations of laser power and travel speed. Here, the mean α is averaged from the measured α values of the entire sample. Ultrasonic C-scan images of samples fabricated by the LAM fabricated using 5 typical parameter combinations is shown in (i)-(v), corresponding to (i)-(v) along the line of travel speed 600 mm/min marked in (A). The XRD patterns are presented on the right side of the ultrasonic C-scan images.

To screen out the optimum process parameters for the LAM of Zr51 BMG with the slightest flaws, we synthesize a parameter library, constituted by a series of rectangular Zr51 BMG samples (40 mm × 18 mm × 3 mm), by the LAM method under continuously changed combinations of laser power and travel speed. The parameter variation within the library ranges from 1200 W to 1650W for laser power and 400 mm/min to 750 mm/min for travel speed. Figure 3(A) shows the mean α value mapping for the BMGs fabricated by the LAM from various combinations of laser power and travel speed. Here, the mean α is averaged from the measured α values of the entire sample. Apparently, the parameter-combination range of laser power spanning from about 1250 to 1350 W along the approximately constant travel speed 600 mm/min yields the lowest α.

To visualize the detail flaw distribution directly, ultrasonic C-scan images of samples fabricated using 5 typical parameter combinations is shown in Fig. 3(i–v), corresponding to (i)-(v) along the line of travel speed 600 mm/min marked in Fig. 3(A). When the laser power is 1200 W, some local regions with strong attenuation α are observed (Fig. 3(i)). These isolated high α regions with an approximately spherical shape may relate to the pores, which result from incomplete re-melting of some local surfaces from the previous layers or the dissolved gas in the molten pool that cannot come out of the surface of the molten pool before solidification due to the high cooling rate^25,26^. These isolated high α regions that induced by the pores can be eliminated by lowering the cooling rate or increasing the laser energy input appropriately. As the laser power increasing to 1300 W, the isolated high α regions indeed reduce obviously, replaced by some long-strip attenuation regions with lower α (Fig. 3(ii)). These long-strip lower α regions may stem from the crystalline regions in the BMG sample fabricated by the LAM. As can be seen from the XRD patterns presented on the right side of the ultrasonic C-scan images, the XRD pattern of sample manufactured at the laser power of 1300 W exhibits a few sharp crystal peaks superimposed on a broad amorphous peak, demonstrating that BMG sample fabricated by the LAM is partially crystallized. During the LAM process, a BMG sample is added layer by layer and a layer is built by overlapping adjacent scan tracks. The original amorphous structure formed in the former track within the overlapping region will crystallize by the laser reheating during the following adjacent track^27^. Then the long-strip attenuated regions are observed to distribute parallel to the laser scan direction between the tracks. Strong evidence supporting the hypothesis that the long-strip attenuated regions are originated from the crystallization in the overlapping regions is the attenuation enhancement with the increasing laser power. As shown inFig. 3(iii), the attenuation of the parallel long-strip attenuated regions becomes more serious as the laser power is further increased to 1400 W. This is because the parallel overlapping regions of the BMG fabricated by the LAM crystallize more severely with the increasing of laser power. In addition, some isolated high α regions associated with the pores reappear in Fig. 3(iii). This is because the turbulence of molten pool becomes more intense as the laser power input is large, thereby entrapping more gas pores^28^. Furthermore, during the rapid melting and solidification process of LAM, the higher energy input also result in a larger residual thermal stress, which would promote the micro-cracks initiating from pores^29^. Thus, some longitudinal high α lines are observed to connect with isolated high α spherical regions in Fig. 3(iii). With gradual increasing the laser power, as shown in Fig. 3(iv) and (v), the attenuations of both horizontal and longitudinal attenuated regions become increasingly severe. Therefore, the laser power of 1300 W and travel speed of 600 mm/min, marked as star in Fig. 3(A), is screened out as the optimal parameter combination for the LAM of Zr51 BMG.

### Verification for the predictability of the high-throughput method

**Figure 4:**
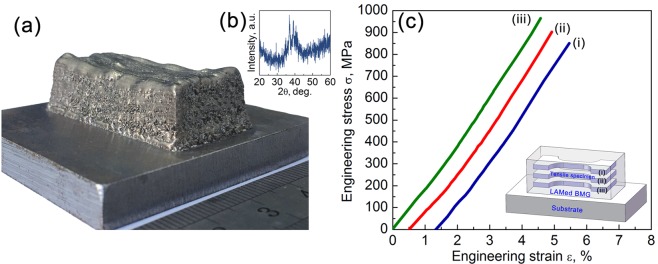
Verification for the predictability of the high-throughput method. (**a**) The macroscopic morphology of the large-sized Zr51 BMG fabricated by the LAM fabricated by the optimal parameter combination of the laser power of 1300 W and travel speed of 600 mm/min, (**b**) The XRD pattern of the cross-section perpendicular to laser travel direction. (**c**) The room-temperature tensile engineering stress-strain curves of the fabricated Zr51 BMG. A schematic of the sampling positions is shown in the inset.

To verify the predictability of our high ultrasonic wave method, the laser power of 1300 W and travel speed of 600 mm/min are chosen as the LAM parameters to fabricate large-sized Zr51 alloy sample with the dimension of 40 mm × 18 mm × 15 mm. The macroscopic morphology of this Zr51 BMG fabricated by the LAM is shown in Fig. 4(a). There are no obvious cracks or defects can be found on the surface of the bulk sample. The XRD pattern of the cross-section perpendicular to laser travel direction is shown in Fig. 4(b), which exhibits some crystalline diffraction peaks superimposing a broad halo peak profile, indicating that a large amount of amorphous phase is still present in the large-sized Zr51 BMG. Tensile test, which is the most direct way to evaluate the impact of flaws, is carried out for the Zr51 BMG fabricated by the LAM. Figure 4(c) displays the room-temperature tensile engineering stress-strain curves of the fabricated Zr51 BMG. A schematic of the sampling positions is shown in the inset of this figure. Clearly, all the three tensile specimens exhibit elastic behavior only, failing without yielding. The fracture stress varies in the range of 850–965 MPa. For comparison, we also measure the tensile properties of the samples fabricated by the other four typical parameter combinations shown in Fig. 3. For the laser power of 1200 W, the fracture stresses are in the range of 807–828 MPa. This lower fracture stress comparing to that of the metallic glass produced by the optimal parameter is due to the relatively severe pore defects. For the laser power larger than 1300 W, the tensile samples fracture with limited stresses, which is induced by micro-cracks originated from large residual thermal stress. In addition, the fracture stresses of the sample fabricated by the optimal parameter is lower than that of the monolithic Zr51 BMG produced by copper mold casting^30^. This probably has to do with the unavoidable pore defects and crystalline phases. Even so, a BMG fabricated by the LAM possessing sufficiently high tensile strength is still noticeable. This is because the huge thermal stress in the LAM process always leads to hidden microcracks, which seriously damage the mechanical properties of deposited BMGs. Only a few works show that the BMGs fabricated by LAM can undergo deformation in compression, but none in tension^7,10,31,32^. Thus, the ultrasonic wave is indeed an efficient and accurate tool to detect the flaws and screen out the optimum process parameters for the LAM of BMGs.

## Conclusion

We have demonstrated a novel high-throughput method that involves ultrasonic wave technique to systematically screen the optimum process parameters for the LAM of Zr51 BMG. A parameter library, constituted by a series of rectangular Zr51 BMG samples, is rapidly fabricated by the LAM method under continuously changed combinations of laser power and travel speed. The ultrasonic attenuation factor α, which is sensitive to the flaws such as pores, cracks and crystalline phases, is used as the monitor to screen the parameters of the BMGs fabricated by the LAM. The laser power of 1300 W and travel speed of 600 mm/min are estimated as the optimum parameter combination for the LAM of Zr51 BMG with the slightest flaws. The amorphous phase dominated microstructure and the sufficiently high tensile strength of the subsequent fabricated large-sized Zr51 BMG sample verify the optimum process parameters.

## Methods

### Material preparation

LAM of Zr51 BMGs was performed with coaxial powder feeding laser solid forming system equipped with a 6000 W fiber laser. Experiments were conducted inside a working chamber, which is filled with argon to keep the oxygen level lower than 10 ppm. The diameter of laser beam was 3 mm. Zr51 metallic powder with a size distribution ranging from 45 to 100 μm was used for laser additive manufacturing process. The powder stored in the powder hoppers was fed through four coaxial nozzles by argon flow and injected into the melt pool created by the laser beam. The powder was delivered into the laser molten pool with the rate fixed at 16 g/min^33^. The 45 steel plates with the dimension of 50 mm × 30 mm × 15 mm were used to act as substrates during laser cladding process. The substrate surfaces were ground with 600 grit SiC papers and thoroughly cleaned in ethanol prior to laser processing. Figure 1 provides a schematic illustration of the overall experimental concept.

### Flaw characterization

Ultrasonic wave testing and X-ray micro computed tomography are performed to evaluate the flaws in the Zr51 BMGs fabricated by the LAM. For ultrasonic wave testing, flaws can be detected from ultrasonic signals discontinuity in the wave path. Ultrasonic measurements are performed using the immersion pulse-echo setup. Both planar and focused transducers with 15 MHz of center frequency were applied. Ultrasonic C-scan image provides quantitatively a two-dimensional view of a specimen in which differences in image contrast result from the flaws interaction with an impinging ultrasonic wave^34^. X-ray micro computed tomography measurement is performed on a TomoScope HV Compact CT machine. The system was equipped with a 225 kV X-ray source with a minimum focal spot size of 3 um and a 1024 × 1024 pixels 16-bit amorphous silicon sensor flat panel detector. The source-to-detector distance was 1100 mm^35^. X-rays are transmitted through the specimen and detected using a flat panel detector. The scanning parameters are as follows, the voltage is 220KV, the current is 300uA, and the scans were done at 50 um resolution, such that the entire sample fits in a single scan volume.

### Material characterization

An XRD equipped with a Cu Kα X-ray source was used to test the phase of manufactured BMG samples. Uniaxial tension tests are performed with an Instron-type machine in the strain rate range of 2 × 10^−4^ s^−1^. The tensile specimens are machined into a dog-bone geometry with dimensions of a gauge length of 6 mm, a width of 2.0 mm, and a thickness of 1.5 mm.
